# Prognostic value of culprit artery double-stranded DNA in ST-segment elevated myocardial infarction

**DOI:** 10.1038/s41598-018-27639-z

**Published:** 2018-06-18

**Authors:** Xiqiang Wang, Dandan Yang, Jing Liu, Xiude Fan, Aiqun Ma, Ping Liu

**Affiliations:** 1 Department of Cardiovascular Medicine, First Hospital of Xi’an Jiaotong University, Xi’an, Shaanxi Province China; 2 Department of Cardiovascular Medicine, Second Affiliated Hospital of Zhejiang University School of Medicine, Hangzhou, Zhejiang Province China; 3 Department of infectious diseases, First Hospital of Xi’an Jiaotong University, Xi’an, Shaanxi Province China; 4 Key Laboratory of Molecular Cardiology, Xi’an, Shaanxi Province China; 5 Key Laboratory of Environment and Genes Related to Diseases (Xi’an Jiaotong University), Ministry of Education, Xi’an, Shaanxi Province China

## Abstract

The double-stranded DNA (dsDNA) which is scaffold of neutrophil extracellular traps (NETs) has been reported to be associated with the occurrence of major adverse cardiovascular events (MACEs) in patients with coronary atherosclerosis. However, the relationship between the dsDNA and the occurrence of MACEs in patients with ST-segment elevated myocardial infarction (STEMI) remains unclear. In this study, 142 consecutive STEMI patients were admitted to medical institutions. Blood from the infarct-related coronary artery (ICA) and peripheral artery (PA) were obtained during percutaneous coronary intervention. Clinical follow-up was performed to analyze the occurrence of MACEs. Patients were divided into low ds-DNA group and high dsDNA group according to the cut-off value of ICA dsDNA. Mean follow-up time was 24.52 months in low dsDNA group and 25.71 months in high dsDNA group. dsDNA in the ICA was significantly higher than in the PA (*p* = 0.038) and Spearman's correlation analysis showed that they were positively correlated (r = 0.758; *p* < 0.01). ICA dsDNA correlated negatively with ST-segment resolution (r = −0.227; *p* = 0.007). The long-term MACEs rate was higher in high dsDNA group than low dsDNA group (23.7 vs. 6.7%, *p* = 0.015). The ICA dsDNA (OR 7.43 95% CI 1.25 to 4.07, *p* = 0.027), Killip class (OR 5.01 95% CI 1.11 to 4.37, *p* = 0.025), BMI (OR 1.36 95% CI 1.06 to 1.7, *p* = 0.016) and white blood cell count (OR 1.27 95% CI 1.03 to 1.57, *p* = 0.024) were independent predictors of the occurrence of MACEs.

## Introduction

Acute ST segment elevation myocardial infarction (STEMI) is the leading cause of death in patients with coronary heart disease^1^. However, the pathogenesis of STEMI is still not well understood. Increasing evidences demonstrated that neutrophil extracellular traps (NETs), which contain chromatin, histones and neutrophil granular, offered a novel role in atherothrombosis^2^. Neutrophils released NETs as signals to promote thrombosis during atherothrombosis^3^.

Recently, the circulating double-DNA which is scaffold of NETs has been reported to be an independent predictor of occurrence of adverse cardiac event and associated with prothrombotic state in coronary atherosclerosis patients^3^. However, to our knowledge, the relationship between dsDNA and the occurrence of MACEs in STEMI patients has not been studied. It has been shown that coronary NETs burden and deoxyribonuclease activity are independently associated with myocardial infarct size and ST-segment resolution^4^. Therefore, we compared the double-stranded DNA (dsDNA) in infarct-related coronary artery (ICA) and peripheral artery (PA) in STEMI patients undergoing primary percutaneous coronary intervention (pPCI) and looked for a threshold of ICA dsDNA predicting occurrence of MACEs.

## Results

### Baseline characteristics

**Figure 1:**
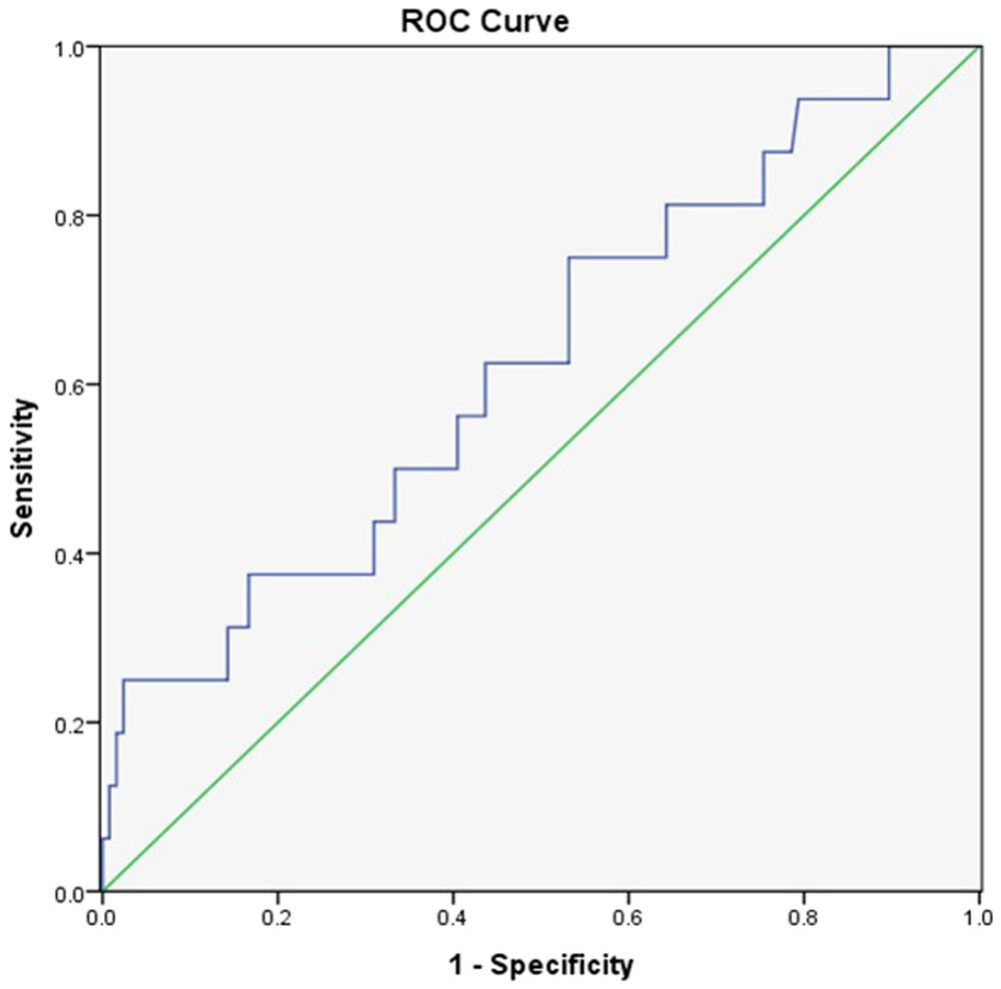
Receiver-operator characteristic curve of the optimal cutoff value of admission dsDNA for predicting the long-term clinical outcomes.

142 STEMI patients (113 men; 79.5%) with the median age of 59 years (min-max, 28–88 years) were studied. All patients underwent coronary angiography (CAG) and PCI. On the basis of the ROC curve analysis, an optimal cutoff value of 0.23 for the ICA dsDNA was selected to predict the major adverse cardiovascular events (sensitivity 75%, specificity 46.2%, area under the curve 0.63) (Fig. 1). According to this criterion, the patients were divided into the following two groups: low ds-DNA group (⩽0.23, n = 45) and high dsDNA group (>0.23, n = 97). Baseline characteristics are presented in Table 1. dsDNA was 0.56 ± 0.34 in the high dsDNA group and 0.16 ± 0.42 in the low ds-DNA group (*p* < 0.001). Patients with a low dsDNA level had higher systolic blood pressure (*p* = 0.003) and diastolic blood pressure (*p* = 0.03), while those with high dsDNA level had higher admission level of Creatine kinase (CK) (*p* = 0.015), Creatine kinase -MB (CK-MB) (*p* = 0.02) and troponin T (*p* = 0.012). The frequency of Killip IV class on admission was lower in the patients with a low ds-DNA level (10.5% vs. 0%).

**Table 1:** Baseline characteristics of patients.

Variable	Low dsDNA group (⩽0.23, n = 45)	High dsDNA group (>0.23, n = 97)	*p*
Age (years)	60.49 ± 11.1	58.07 ± 12.93	0.3
Males	32 (76.2%)	81 (83.5%)	0.2
BMI (kg/m^2^)	24.46 ± 3.06	24.07 ± 2.76	0.457
Smoking	19 (46.3%)	35 (37.7%)	0.30
Medical history			
Hypertension	15 (36.6%)	35 (37.7%)	0.98
Diabetes mellitus	7 (17%)	21 (22.1%)	0.75
Myocardial infarction	0	8 (5.6)	0.13
Revascularization	0	10 (10.5%)	0.07
Chronic heart failure	0	2 (2.1%)	0.87
Cerebrovascular disease	2 (4.9%)	2 (2.1%)	0.75
Admission heart rate (beats/min)	80.17 ± 13.68	77.45 ± 19.44	0.42
Systolic blood pressure (mmHg)	135.61 ± 19.35	123.46 ± 22.75	0.003
Diastolic blood pressure (mmHg)	83.88 ± 12.05	78.02 ± 15.09	0.03
Onset to admission (h)	5.43 ± 3.19	5.39 ± 3.33	0.95
Admission to the PCI (min)	52.5 ± 3.45	51.92 ± 10.49	0.94
ds-DNA (ug/ml)	0.16 ± 0.42	0.56 ± 0.34	<0.001
TAT complexes (ng/ml)	22.94 ± 18.60	24.30 ± 17.04	0.68
STR (%)	69.01 ± 0.25	64.6 ± 0.22	0.29
Troponin (ng/mL)	0.57 ± 0.89	1.39 ± 2.58	0.012
Creatine kinase -MB (U/L)	76.3 ± 111.85	132.8 ± 156.12	0.02
Peak creatine kinase -MB (U/L)	3619.12 ± 2530.17	3965 ± 2467.4	0.46
Creatine kinase (U/L)	761.69 ± 1701.71	1684.95 ± 2513.04	0.015
Peak creatine kinase (U/L)	3619.12 ± 2530.17	3965.02 ± 2467.44	0.46
AST (U/L)	191.57 ± 161.53	213.81 ± 147.43	0.44
Total cholesterol (mmol/L)	4.26 ± 1.08	3.94 ± 1.07	0.11
Low-density lipoprotein(mmol/L)	2.48 ± 0.77	2.36 ± 0.75	0.39
High-density lipoprotein(mmol/L)	0.98 ± 0.25	0.95 ± 0.21	0.59
Triglycerides (mmol/L)	1.51 ± 0.87	1.94 ± 2.49	0.28
Hemoglobin (g/L)	140.95 ± 20.14	144.47 ± 22.41	0.39
Platelet count (10^9^/L)	217.12 ± 70.38	204.46 ± 76.36	0.37
WBC (10^9^/L)	11.05 ± 3.11	11.44 ± 3.97	0.58
Neutrophil (10^9^/L)	10.92 ± 12.38	11.97 ± 14.16	0.09
Monocyte (10^9^/L)	0.39 ± 0.18	0.50 ± 0.59	0.22
N-terminal pro-Brain natriuretic Peptide (pg/ml)	1857.44 ± 1957.03	1011.46 ± 1790.71	0.68
Admission blood glucose (mmol/L)	7.46 ± 3.22	7.18 ± 3.13	0.64
Glycosylated hemoglobin	5.93 ± 1.11	5.91 ± 1.32	0.91
Homocysteine (umol/l)	24.3 ± 16.6	24.17 ± 15.82	0.97
LVEF (%)	46 ± 4.58	42.5 ± 13.53	0.69
LVFS (%)	29.5 ± 2.12	24.5 ± 2.12	0.14
Killip class on admission			0.07
I	34 (82.9%)	74 (77.9%)	
II	4 (9.8%)	9 (9.5%)	
III	3 (7.3%)	1 (1.1%)	
IV	0	10 (10.5%)	
Number of narrowed coronary vessels	2.13 ± 0.88	2.35 ± 1.23	0.29
Culprit lesion (coronary)			0.23
Right	12 (30%)	33 (35.9%)	
Left anterior descending	27 (67.5%)	46 (50%)	
Left circumflex	1 (2.5%)	8 (8.7%)	
Left main	0	3 (3.3%)	
Others	0	2 (1.4%)	
TIMI flow grade pre-PCI			0.48
0	21 (52.5%)	62 (66.7%)	
1	2 (7.5%)	3 (3.2%)	
2	4 (10%)	7 (7.5%)	
3	12 (30%)	21 (22.6)	
Stent placement number	1.54 ± 0.82	1.67 ± 1.06	0.5
Follow-up time (month)	24.52 ± 10.64	25.71 ± 10.19	0.52
Drugs after discharge			
Aspirin	40 (100%)	88 (97.8%)	0.34
Clopidogrel	40 (100%)	89 (98.9%)	0.82
Statin	40 (100%)	89 (98.9%)	0.82
Angiotensinconverting enzyme Inhibitors	31 (90%)	69 (80%)	
Angiotensin II receptor blocker	1 (2.5%)	0	0.29
Beta-blocker	40 (100%)	90 (100%)	0.57

BMI, body mass index; TIMI, Thrombolysis in Myocardial Infarction; PCI, percutaneous coronary intervention; LVEF, left ventricular ejection on fraction; LVFS, left ventricular fractional shortening; TAT, thrombin-antithrombin; STR: ST-segment resolution; WBC, white blood cell.

### Double-stranded DNA in infarct-related coronary artery and peripheral artery

**Figure 2:**
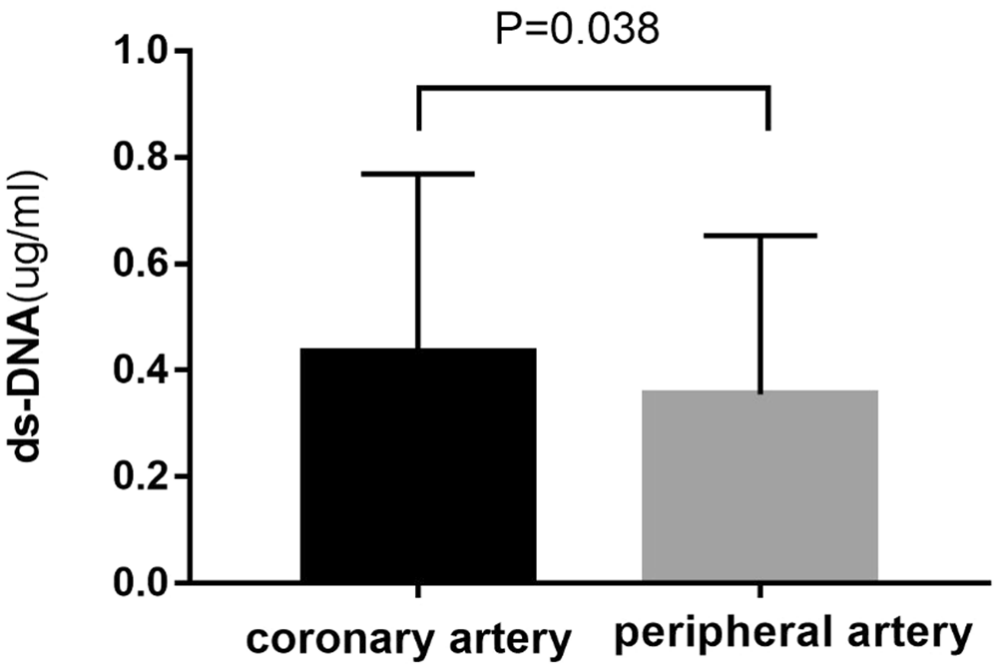
Coronary artery dsDNA vs peripheral artery dsDNA; dsDNA indicates double-stranded DNA.

Quantitative analysis with the fluorescent instrument showed that the dsDNA in the infarct-related coronary artery was dramatically higher than in the peripheral blood plasma (*p* = 0.038) (Fig. 2). Spearman’s correlation analysis showed that dsDNA contents in the ICA and PA were positively correlated (r = 0.758, *p* < 0.01) and the ICA dsDNA concentrations were negatively correlated with STR (r = -0.227; *p* = 0.007).

### Survival analyses

Mean follow-up time was 24.52 months in low dsDNA group and 25.71 months in high dsDNA group. The long-term MACEs rate (23.7 vs. 6.7%, *p* = 0.015) was higher in patients with a high level of ds-DNA than those with a low level of dsDNA, the long-term cardiac mortality rate was 4.1 vs. 0% and the all-cause mortality rate was 8.2 vs. 6.7%, although the differences were not statistically significant (*p* = 0.40 and 0.74) as shown in Table 2.

**Table 2:** Long-term of MACEs.

Variable	Low ds-DNA group (n = 45)	High ds-DNA group (n = 97)	*P*
All-cause mortality	3 (6.7%)	8 (8.2%)	0.74
Cardiac mortality	0	4 (4.1%)	0.40
Recurrence of PCI	0	4 (4.1%)	0.40
Recurrence of ACS	0	3 (3.1%)	0.57
Ischemic stroke	0	4 (4.1%)	0.40
MACEs	3 (6.7%)	23 (23.7%)	0.015

MACEs, major adverse cardiovascular events; PCI, percutaneous Transluminal Coronary Intervention; ACS, acute coronary syndrome.

**Figure 3:**
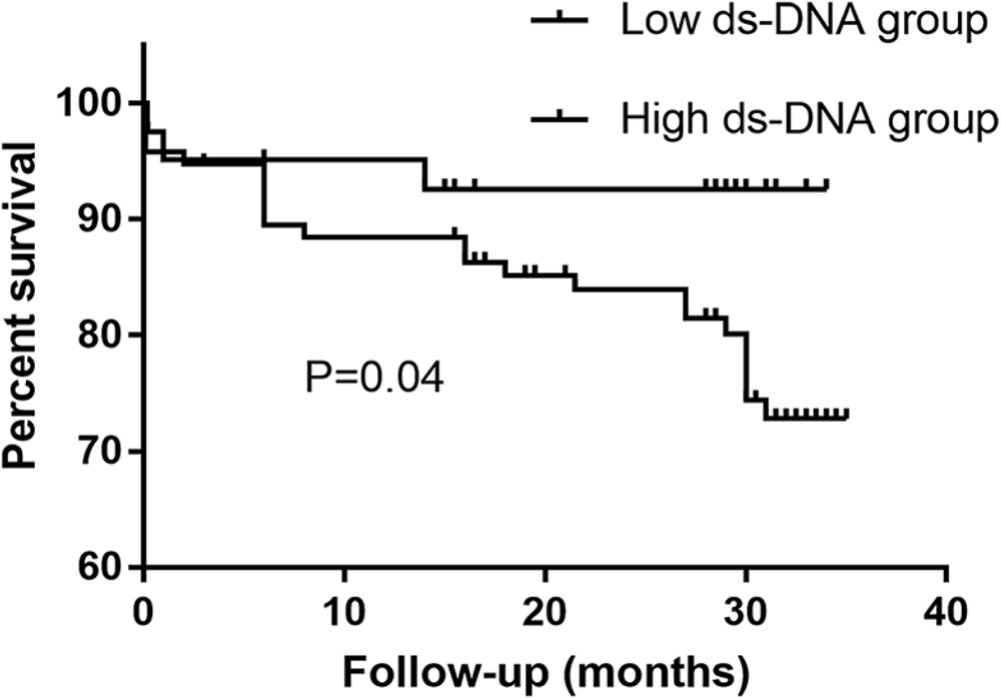
Survival curve in terms of the long-term clinical outcomes according to ds-DNA groups in the entire patient cohort.

Take the MACEs as a composite study endpoint, Kaplan-Meier survival curve was performed. As shown in Fig. 3, a high dsDNA level was associated with the occurrence of MACEs (*p* = 0.04). In the multivariate analysis, the ICA dsDNA (OR 7.43 95% CI 1.25 to 4.07, *p* = 0.027), Kilip class (OR 5.01 95% CI 1.11 to 4.37, *p* = 0.025), BMI (OR 1.36 95% CI 1.06 to 1.7, *p* = 0.016) and white blood cell count (OR 1.27 95% CI 1.03 to 1.57, *p* = 0.024) were independent predictors of MACEs (Table 3).

**Table 3:** Multivariable predictors of MACEs.

Variable	Adjusted OR	95% CI	*P*
Killip class	5.01	1.11 ∼ 4.37	0.025
ds-DNA	7.43	1.25 ∼ 4.07	0.027
BMI	1.36	1.06 ∼ 1.7	0.016
WBC	1.27	1.03 ∼ 1.57	0.024

BMI, body mass index; CI, confidence interval; MACEs, major adverse cardiovascular events; OR, odds ratio; WBC, white blood cell; Adjusted for: age, BMI, gender, smoking, hypertension, diabetes, hyperlipidemia, Killip-class on admission, heart rate on admission, systolic blood pressure on admission, percentage of ST-segment resolution, thrombin-antithrombin, diastolic blood pressure on admission, white blood cell count, neutrophil, glutamic oxalacetic transaminase, CK and CK-MB on admission, troponin T on admission, dsDNA in ICA and PA, LDL and HDL.

## Discussion

The principal findings of this observational prospective clinical study are as follows: a) dsDNA is significantly increased in ICA compared to PA; b) patients with a higher dsDNA level shows an increased long-term adverse cardiac event rate; c) Higher ICA dsDNA level is a predictor of long-term clinical outcomes in patients with STEMI undergoing PCI. Because the test of dsDNA is inexpensive and simple to determine, dsDNA could potentially serve as a predictor for the adverse cardiac events in patients with STEMI undergoing PCI.

Neutrophils and leukocytes are first-line defense cells in human innate immune system^5^. In the past decades years, inflammatory response had been found to be associated with the increased risk of atherosclerosis and that could turn into acute myocardial infarction, neutrophils played a key role in this process^6^. Numerous studies demonstrated that elevated leukocyte count and its subtype were associated with the increased risk of adverse cardiac events in acute myocardial infarction^7–9^. Despite highlighting the adverse effect of neutrophils and leukocytes in the early phase of acute myocardial infarction on clinical outcome, it remains unclear whether elevated systemic neutrophils are directly impact clinical outcomes. In 2004, Brinkmann, V. *et al*. first observed that neutrophils form NETs via release granule proteins and chromatin the process of which was named extracellular trap formation (ETosis), a death program other than necrosis or apoptosis that may account for the role of neutrophils in acute myocardial infarction. NETs are composed of decondensed DNA backbone and antimicrobial proteins which providing an extracellular scaffold to prevent microorganisms from spreading and kill bacteria^10^. In addition to its microbicidal function, NETs was also found to be connected with other diseases such as venous thrombosis, sepsis, SLE and cancer^11–14^. In addition, a study demonstrated that in severe coronary atherosclerosis patients, the elevated levels of dsDNA and chromatin were independently connected with the occurrence of MACEs and prothrombotic state^3^.

In our study, we demonstrated that the level of dsDNA increased significantly in ICA compared to PA, which was in agreement with the recently publications by Mangold and A. stakos^2^. Extracellular dsDNA had been identified as a maker of NETs and the scaffold of NETs, which could be degraded by deoxyribonuclease I (DNase-I)^15^. Recently, Lan Ge and his coworkers found that DNase-based regimen could reduce NET density and improve coronary microvasculature patency and attenuate infarct size in an ischemia-reperfusion mouse model^2^. The polymorphism of Gln222Ary in the DNase-I gene was independently connected with a higher rate of myocardial infarction^16^. *In vivo*, DNase could degrade human coronary thrombi and accelerate lysis^4^. These data suggest that the dsDNA which is the scaffold of NETs, could serve as a potential therapeutic target for STEMI patients.

In the present study, we observed that high dsDNA group had higher levels of creatine kinase, creatine kinase MB, troponin T and an increased frequency of killip IV class. Myocardial enzyme and troponin are associated with infarct size. The killip class is used to assess the damage of the cardiac function in STEMI patients. Thus, our findings demonstrated that a higher dsDNA was associated with larger infarct size. We speculate that this association caused by neutrophils. Activated neutrophils via NETosis release NET, extracellular dsDNA and protein composed of NETs scaffold, which is a weblike structure^10^. This weblike structure play an important role for tissue factor functionality in STEMI patients^2^ and activated neutrophils play a key role in this process. In agreement with above theories, a lot of studies showed an association between culprit site neutrophils and enzymatic infarct size^17^. Distelmaier and coworkers also found that patients with increased culprit site neutrophil accumulation had higher mortality and larger infarct sizes^18^. Measuring the creatine kinase level and using computed tomographic examination, Dragu *et al*. reported that peripheric neutrophil count directly reflected the extent of myocardial damage^19^. A study published by Mangold, underlined the pivotal role of dsDNA by demonstrating an association between coronary NET burden and ST-segment resolution. In keeping with our findings, they demonstrated that coronary NET burden positively correlated with enzymatic and infarct size measured by cardiac magnetic resonance^4^. Our study expanded the sample size, reconfirmed the previous studies conclusion and identified dsDNA as a potential therapeutic target for STEMI patients.

Recently, NETs and its related structures have emerged as contributors to coronary atherosclerotic heart disease^2–4,20^. Besides, NETs were identified in culprit artery of human atherosclerotic plaque and our observational prospective study was the first experiment to examine the relationship between the dsDNA and the long-term clinical outcomes in STEMI patients undergoing PCI, we demonstrated that high level of dsDNA in ICA was an independent predictor of adverse cardiac events. But the pathogenesis of NETs in STEMI remains unclear and it is unclear whether an increased dsDNA is a marker or a predictor of adverse cardiac events. A current study showed that the structure of NETs served as key indicators of cardiovascular inflammation and it was confirmed in a murine model of atherosclerosis that NETs primed macrophages for cytokine production. Then, the interaction of neutrophils and cytokine drove sterile inflammation in atherosclerosis^21^. Using the coronary sampling of STEMI patients, neutrophils in infarct-related coronary artery releasing NETs and exposing functional tissue factor. The exposed tissue factor on NETs was capable of inducing thrombin generation and platelet activation mediated by thrombin. They also confirmed that the NETs scaffold integrity was important for tissue factor functionality^2^. Taken together, we speculate that NETs trigger thrombotic vascular occlusion in acute myocardial infarction patients by the release of cytokine, exposing functional tissue factor, weblike structure formation and thrombin generation. In order to better understand the precise role of NETs in thrombus formation among STEMI patients, the role of cytokine release and dsDNA must be explored more precisely in future studies. Although our observational prospective study does not show causality, the dsDNA appears to be related to larger infarct size and the occurrence of adverse cardiac events.

The long-term cardiac mortality rate, the all-cause mortality rate, recurrence of PCI, recurrence of ACS and ischemic stroke were significantly higher in patients with a high ds-DNA than in those with a low dsDNA, but the differences were not statistically significant. The following reasons should be taken into account. First, the sample size of STEMI patients in this study is small. Second, the trails duration maybe too short to catch the significant difference in these observation target. In conclusion, larger studies with a longer follow-up are needed to better assess the sensitivity and specificity of dsDNA to predict the occurrence of MACEs. It also remains of interest to further test whether levels of the dsDNA predict adverse outcomes. Because the test of dsDNA is inexpensive and easily available, a broader use may be considered to identify patients at higher risk and the NETs maybe a novel therapeutic target for STEMI patients.

## Methods

### Study population

From June 1, 2015 to December 31, 2015, 142 consecutive patients with STEMI admitted to medical institutions within 12 hours after symptom and for pPCI immediately were recruited in this prospective cohort study. Patients on valvular heart disease, history of atrial fibrillation and dilated cardiomyopathy, with blood disorders, thyroid disease, severe hepatic or renal dysfunction, recently with the wounded as well as ongoing chronic inflammatory, autoimmune (rheumatoid arthritis, systemic lupus erythematosus, ulcerative colitis), or malignant diseases and the patients who disagree to complete this study were excluded. These patients who admitted to medical institutions were transferred immediately at the catheterization laboratory and received low molecular weight heparin and aspirin plus clopidogrel oral loading. This prospective cohort study was in compliance with the Declaration of Helsinki and was approved by the Ethics Committees of First Hospital of Xi’an Jiaotong University. All patients were given written informed consent.

Demographic variables, traditional cardiovascular risk factors, clinical variables, laboratory values were collected after PCI. ST-segment resolution (STR) was calculated by measuring STE at the J point in respective leads (for anterior infarction: I, aVL, and V1–V6; for inferior infarction: II, III, aVF, V5, and V6) in the index-electrocardiography and 30 minutes after pPCI. The ratio of the ST-elevation (STE) sum was calculated and expressed in percent^22^. After discharge, all of the patients were given the standard drug treatment according to the ACC/AHA guidelines for diagnosis and treatment of STEMI^23^.

### Immunofluorescence

Blood from the ICA and PA were obtained during primary percutaneous revascularization in patients with STEMI. The samples used for isolation of neutrophils and plasma within 30–60 min after the transfer to the catheterization laboratory. dsDNA, as an indirect marker of NET formation, was established using Sytox Green nucleic acid stain (5 μM, Invitrogen) in a fluorescence microplate reader at 488 nm excitation and 525 nm emission.

### Study Endpoint and Follow-up

The composite study endpoint was the occurrence of major adverse cardiovascular events (MACEs) including all-cause mortality, cardiac mortality, recurrence of acute coronary syndrome, recurrence of PCI, coronary artery bypass grafting (CABG), ischemic stroke and hemorrhagic stroke. Patients were regularly examined by their cardiologists. Outpatient visits, emergency room visits and hospital admissions were recorded in their electronic patient records.

### Statistical Analysis

Continuous variables were expressed as mean ± SD and were compared using Student t test or Mann-Whitney rank sum test as appropriate. Categorical data were expressed as numbers and percentages and were compared using Pearson’s chi-square test (χ^2^). The relationship between the coronary artery dsDNA and the peripheral artery dsDNA were examined using Spearman’s rank correlation test. We used receiver-operator characteristic (ROC) curve analysis to determine the optimal cutoff value of the coronary artery dsDNA for predicting MACEs. A stepwise multivariate logistic regression analysis was performed to identify the independent risk factors of MACEs during the follow-up. The potential covariates included in this regression model were the age, BMI, gender, smoking, hypertension, diabetes, hyperlipidemia, Killip-class on admission, heart rate on admission, systolic blood pressure on admission, percentage of ST-segment resolution, thrombin-antithrombin, diastolic blood pressure on admission, white blood cell count, neutrophil, glutamic oxalacetic transaminase, CK and CK-MB on admission, troponin T on admission, dsDNA in ICA and PA, LDL and HDL. Cumulative survival curve for MACEs was constructed using the Kaplan-Meier method. *p* value < 0.05 was considered statistically significant. Statistical analyses were performed using IBM SPSS Statistics 20.0.0 (SPSS Inc, IL, USA).
